# Self-perceived competence and readiness of undergraduate physiotherapy students in treating patients with spinal cord injury

**DOI:** 10.4102/sajp.v82i2.2316

**Published:** 2026-04-30

**Authors:** Mokgadi K. Mashola, Tamsyn Bauermeister, Clare N. Urio, Alexandra Michael, Rabelani Masindi, Kgaogelo M. Mpe, Lorraine Dippenaar, Karien Mostert

**Affiliations:** 1 Department of Physiotherapy, Faculty of Health Sciences, University of the Witwatersrand, Johannesburg, South Africa; 2 Department of Physiotherapy, Faculty of Health Sciences, University of Pretoria, Pretoria, South Africa

**Keywords:** clinical education, competence, readiness, self-perception, spinal cord injury

## Abstract

**Background:**

Physiotherapy management is a necessary core aspect in spinal cord injury (SCI) rehabilitation, and undergraduate preparation for adequate application is imperative.

**Objectives:**

To determine physiotherapy students’ self-perceived competence and readiness in treating patients with SCI.

**Method:**

This study included 48 fourth-year students enrolled in a Bachelor of Physiotherapy degree in South Africa. Students completed a Qualtrics^XM^ online survey consisting of 32 questions self-compiled from the Assessment of Physiotherapy Practice (APP) tool and the Dundee Ready Education Environment Measure (DREEM) questionnaire to determine self-perceived competence and readiness to treat SCI patients. Descriptive (frequencies and means) and inferential data (independent *t*-test and Fisher’s exact test) were analysed using SPSS v26 at a 0.05 level of significance.

**Results:**

Students reported 70% self-perceived competence and 73% readiness to treat SCI patients. Theoretical knowledge was perceived as adequate (*p* < 0.01), and students who completed their SCI clinical block perceived themselves as more competent in treating patients with SCI (*p* < 0.01). They were able to clinically reason and adequately differentiate between diagnoses (*p* < 0.01) and safely perform treatment techniques (*p* < 0.01).

**Conclusion:**

Students who completed their SCI clinical block had higher self-perception of competence to differentially diagnose SCI and to treat SCI patients safely, compared to those who had not.

**Clinical implications:**

Hands-on experience of SCI patient care is encouraged before SCI clinical block rotation. In cases where there are limited clinical placements, universities are recommended to augment written case scenarios with finite supervised patient exposure.

## Introduction

Over 15 million people are living with spinal cord injury (SCI) (WHO 2024), and physiotherapy management is essential in their rehabilitation (Glinsky & Harvey 2024). Physiotherapy management after SCI refers to restoring function and mobility to affected individuals and can include goal setting, optimising functional activities and preventing secondary health conditions (Harvey 2016). Physiotherapists provide one of the core interventions in SCI rehabilitation, and physiotherapy SCI education is imperative to ultimately give people with SCI (PWSCI) quality of care during their rehabilitation phase. Universities are challenged with the increasing demands of work-ready graduates who have a broad knowledge of chronic conditions, long-term illnesses and promoting health and wellness (Barradell 2017). Most physiotherapy curricula include practice-based education and clinical-based learning, which both have been a highlight in learning experiences, as they involve knowing, doing and being, and are seen to enhance students’ skills during clinical education experiences (Barradell 2017). These enhanced skills are of particular importance, as SCI education requires clinical exposure.

Spinal cord injury education of students involves a sound theoretical foundation in order to effectively incorporate physiotherapy theory into clinical practice to treat patients with SCI (Rossettini et al. 2017). Spinal cord injury rehabilitation programmes focus on the psychosocial, biological, social and mental health aspects of the affected individual. Rehabilitation of PWSCI is considered an advanced skill in physiotherapy education and is taught to senior students (third and fourth year) in South Africa and beyond our borders (Hossain et al. 2015). The teaching content covers the major areas in SCI, intending to equip students with the competency to adequately manage PWSCI. Competency is the ability of an individual to apply or utilise previously acquired knowledge, skills and abilities to perform tasks to achieve the desired results in a professional setting (Chouhan & Srivastava 2014). The competency of physiotherapy students may specifically relate to the effectiveness of learning and skill transfer in an educational environment and how it is applied in a clinical environment. To be clinically competent, students must function safely and effectively as entry-level healthcare practitioners, providing optimal, comprehensive and ethical patient-centred care (World Physiotherapy 2021). Dahl-Michelsen (2014) reported that the extent and precision with which students transfer their knowledge into clinical practice is a direct reflection of the lecturer’s skills and knowledge, suggesting that students are more likely to attain information as implicit and explicit knowledge, which makes them competent in a clinical setting. However, this finding may also suggest that students are unaware of their level of competency to treat a patient with SCI in the clinical setting, which links to an inadequate self-perception of their abilities.

A student’s perception of their academic environment can also influence their learning experience and ability to learn (Bakhshialiabad, Bakhshi & Hassanshahi 2015). Determining students’ perceptions of their competence and readiness for clinical practice can be an informative approach to researching the specific curricula that influence these perceptions, in turn, influence the effectiveness of learning in an environment (Coelho & Moles 2015). Detailed learning by physiotherapy students can influence the way they perceive the work being taught as well as the effectiveness in achieving student learning outcomes (Holdsworth, Skinner & Delany 2016). This learning may directly relate to the students’ readiness to treat PWSCI in a clinical setting. The inverse of this was researched by Lo et al. (2017), who highlighted the lecturers’ perspectives of students in a clinical setting and found that the students lacked clinical skills and competence to practise. This possibly links to perspectives of both the lecturers and students, which can be seen as a discrepancy in the education of physiotherapy students (whereby the problems and gaps in the education may not be addressed). This suggests that lecturers may feel that the work is being taught adequately, but the students are not performing well clinically, or that the students feel that the work is being taught inadequately to prepare them for a clinical setting. Physiotherapy education in SCI rehabilitation in universities globally plays an important role in the perception of the readiness of the undergraduate students to assess, treat and rehabilitate PWSCI. Palmgren et al. (2014) report a generally positive perception among undergraduate physiotherapy students of the educational environment and educational programme provided to them. Talberg and Scott (2014) similarly found that physiotherapy students perceived a high level of readiness for clinical practice. Furthermore, academics and clinicians believe that senior physiotherapy students in South Africa are able to demonstrate appropriate clinical skills (Komati, Prince & Korkie 2025). There is, however, a paucity of South African literature on students’ self-perceived competence and readiness in SCI-clinical care, and this study aims to bridge this gap.

## Research methods and design

This study employed a quantitative cross-sectional study design to collect the relevant competence and readiness information from the students during the same point in time, as supported by Brink, Van der Walt and Van Rensburg (2018). The Bachelor of Physiotherapy degree (also known as the Bachelor of Science in Physiotherapy degree) is offered in only eight universities in South Africa. Specifically, these institutions include the University of the Witwatersrand, University of Pretoria, Sefako Makgatho Health Sciences University, University of the Free State, University of KwaZulu-Natal, University of Cape Town, University of the Western Cape and Stellenbosch University. The fourth-year class of 2020 was recruited through their year coordinators and was sent the Qualtrics^XM^ survey link from March 2020 to June 2020 to participate in the study. Students did not need to have completed an SCI rotation before completing the survey.

A non-probability total population sampling approach was used, where all consenting fourth-year physiotherapy students studying at the above-mentioned universities in South Africa were included in the study. All registered undergraduate fourth-year physiotherapy students (regardless of nationality, age, sex, or race) were invited to participate voluntarily in the survey. No eligible student was excluded from participating in the study.

Data were collected using a five-point Likert scale survey developed by compiling various questions from the Assessment of Physiotherapy Practice (APP) tool and the Dundee Ready Education Environment Measure (DREEM) questionnaire, which focused on self-perception, competence, readiness, knowledge acquisition and knowledge application. The Likert scale was scored as follows: 1 = strongly disagree, 2 = disagree, 3 = neutral, 4 = agree and 5 = strongly agree. The APP tool specifically measures professional behaviour, assessment, communication, analysis, intervention, planning, risk management and evidence-based practice (Dalton, Davidson & Keating 2011) while the DREEM questionnaire measures students’ perception of teaching, teachers, own academic perception, atmosphere and their social self-perception (Roff et al. 1997). These measures were used to establish a benchmark standard to allow students to self-assess, which can assist in the development of more accurate self-perceived competency levels. Three experts were invited to participate in the pilot study to determine face validity, and their independent feedback was used to make changes to the survey. The experts consisted of three academics, one who researched undergraduate student competence and two who had previously tested psychometric properties of tools used in SCI clinical care. The final tool used in this study still requires psychometric testing for reliability and validity.

A pilot study including ten fourth-year students from the University of Pretoria was conducted to determine the clarity of the questions and the time it takes to complete the survey and also to determine which questions to remove and which questions to add in the survey. Additionally, the pilot study assessed whether all students were able to access the survey link and whether all questions were displayed correctly. The 10-min survey was easily accessible, and the questions were understandable. Data from the pilot study were not included in the main study.

Data were analysed using descriptive and inferential statistics in SPSS version 26. Descriptive statistics, such as mean, standard deviation (s.d.), frequencies and percentages, were used. Inferential statistics, such as the independent *t*-test, were used to compare differences between students who had completed an SCI rotation and those who had not at the time of data collection. The Fisher’s exact test was used to determine relationships between demographic data and self-perceived competence and readiness to treat PWSCI. All testing was done at a 0.05 level of significance. Data captured in this study are stored at the University of Pretoria’s Physiotherapy Department until 2035, as per the research ethics committee requirements.

### Quality control

To enhance the rigour of this study, measures of reliability and validity were conducted. Reliability measures whether there exist internal stability and consistency among the test scores of participants (Heale & Twycross 2015). The survey asked questions based on self-perceived competence and readiness. To test whether the survey would produce stable and consistent results, the test–retest method was used as a component of our pilot study. The pilot study was not anonymous, and the same 10 participants in the pilot study who completed the survey were asked to complete it again within a 10-day interval. The participants’ two responses were compared using a paired *t*-test, and the responses showed consistency with no significant differences noted. Face validity was employed to determine whether the survey accurately measured what it intended to measure (Heale & Twycross 2015). Three independent experts, comprising three academics, two in the field of SCI and one in the musculoskeletal field, with expertise in quantitative research, reviewed the questionnaire and gave recommendations, which were all incorporated into the final questionnaire.

### Ethical considerations

This study received ethical approval from the Faculty of Health Science Research Ethics Committee of the University of Pretoria (approval number: 773/2019). Permission to distribute the survey to students was obtained from the Head of the Physiotherapy Department at each respective university. After permission had been granted, the survey link was uploaded onto the university’s specific student communication platform and the class WhatsApp group. Informed consent was obtained by completing the online questionnaire, and confidentiality was maintained as responses were anonymous. There was no financial cost involved for participants to participate in the study, and participants were not compensated for their participation.

## Results

All eight universities received the invitation to participate in the study. One university did not respond, one required the study to repeat a similar 3-month ethical process and three universities stopped communication before accepting participation in the study. Three universities consented, with a total of 48 students responding to the questionnaire. The University of Pretoria had a 73% response rate, the University of the Western Cape 12% and the University of the Witwatersrand 15%. All 48 students responded to the questions regarding the demographic section. Three out of the 48 students did not respond to Section B of the questionnaire, and these questions were reported as ‘missing’.

### Demographic information

Table 1 reflects the demographic information collected from participants. The mean age in this study was 22.23 (s.d. 1.59) years, and there were more female students than male students (85% vs 15%). None of the students had repeated either the third or fourth year, and all students had completed the SCI theory component before the data were collected. The majority of students (94%) did not have prior experience with PWSCI, and 52% had not completed an SCI clinical block at the time of data collection. The most recommended resource used by students was research articles (33.3%), followed closely by lecturers’ notes (31.3%) and websites (31.3%). Textbooks were only recommended by 4.2% of students.

**TABLE 1:** Demographic information.

Variable	Category	*n*	%
Age (years)	21	18	37.5
	22	15	31.2
	23	9	18.8
	24	3	6.3
	25	2	4.2
	30	1	2.1
Sex	Female	41	85.4
	Male	7	14.6
University	University of Pretoria	35	72.9
	University of the Western Cape	6	12.5
	University of the Witwatersrand	7	14.6
Do participants have a previous qualification?	Yes	2	4.2
	No	46	95.8
Have participants completed an SCI-related course prior?	Yes	1	2.1
	No	47	97.9
Do participants have experience with PWSCI?	Yes	3	6.3
	No	45	93.8
Have participants completed their SCI theory?	Yes	48	100.0
	No	0	
In which year was SCI taught to the participants?	2nd	4	8.3
	3rd	37	85.4
	4th	7	14.6
Most used resources by the participants	Textbook	2	4.2
	Research articles	16	33.3
	Lecturer’s notes	15	31.3
	Websites	15	31.3
Frequency of resources used by participants	Never	1	2.1
	Occasionally	43	91.7
	Every day	4	8.3
Participants currently repeating the year?	None	48	100.0
Participants have done an SCI clinical block to date	Yes	23	47.9
	No	25	52.1
Participants have treated a PWSCI to date	Yes	36	75.0
	No	12	25.0
At which level did the participants complete the SCI clinical block?	Not yet completed	15	31.3
	3rd year	21	43.8
	4th year	12	25.0

SCI, spinal cord injury; PWSCI, people with SCI.

**FIGURE 1:**
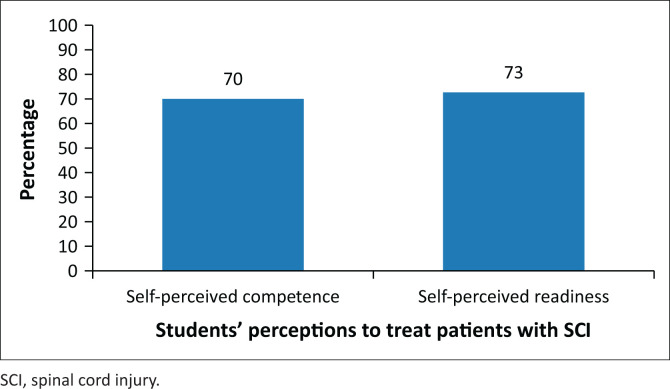
The self-perceived competence and readiness of students.

The mean percent for self-perceived competence was 70% (s.d. 22.03) and 73% (s.d. 21.42) for self-perceived readiness (Figure 1).

Table 2 depicts the frequency and percentage for each question, with many students strongly agreeing that they are more ready for the clinical block when exposed to treating a patient with SCI before the block (75%). Most students agreed that they can safely and appropriately perform treatment techniques on PWSCI, as well as conduct objective assessments on PWSCI (50%, respectively).

**TABLE 2:** Competence and readiness questions.

Question	Strongly disagree		Disagree		Neutral		Agree		Strongly agree		Missing	
	*n*	%	*n*	%	*n*	%	*n*	%	*n*	%	*n*	%
1. Perceived theoretical knowledge in SCI to manage or treat PWSCI.	1	2.1	4	8.3	6	12.5	22	45.8	12	25.0	3	6.3
2. Perceived ability to apply theoretical knowledge in SCI practice.	0	0.0	3	6.3	10	20.8	21	43.8	11	22.9	3	6.3
3. Perceived ability to deal with the range of medical conditions that may be regarded or classified as SCIs.	0	0.0	3	6.3	17	35.4	19	39.6	6	12.5	3	6.3
4. Perceived ability to use knowledge of anatomy, physiology and clinical medicine in clinical reasoning required to treat PWSCI.	0	0.0	4	8.3	11	22.9	25	52.1	5	10.4	3	6.3
5. Perceived ability to safely and appropriately perform treatment techniques on PWSCI.	1	2.1	2	4.2	12	25.0	24	50.0	6	12.5	3	6.3
6. Perceived ability in knowledge of treatments that can be detrimental to specific patients if used inappropriately.	0	0.0	2	4.2	15	31.3	23	47.9	5	10.4	3	6.3
7. Perceived ability to perform subjective assessments on PWSCI.	0	0.0	0	0.0	8	16.7	16	33.3	21	43.8	3	6.3
8. Perceived ability to perform objective assessments on PWSCI.	0	0.0	1	2.1	11	22.9	24	50.0	9	18.8	3	6.3
9. Perceived ability in interpreting assessment findings in relation to PWSCI.	0	0.0	4	8.3	11	22.9	22	45.8	8	16.7	3	6.3
10. Perceived ability to interpret investigative findings in relation to PWSCI.	0	0.0	5	10.4	12	25.0	20	41.7	8	16.7	3	6.3
11. Perceived ability to prioritise PWSCI problems after conducting an assessment.	0	0.0	2	4.2	16	33.3	19	39.6	8	16.7	3	6.3
12. Perceived ability to identify and set appropriate short- and long-term goals for a PWSCI.	0	0.0	5	10.4	15	31.3	20	41.7	5	10.4	3	6.3
13. Perceived readiness for the SCI clinical block after using the prescribed textbook to prepare.	1	2.1	13	27.1	18	37.5	8	16.7	5	10.4	3	6.3
14. Perceived readiness for SCI clinical block after using online learning resources to prepare.	0	0.0	5	10.4	6	12.5	25	52.1	9	18.8	3	6.3
15. Perceived readiness for SCI clinical block after being assessed on skills using a theoretical patient.	1	2.1	7	14.6	10	20.8	18	37.5	9	18.8	3	6.3
16. Perceived readiness for SCI clinical block after watching videos of a patient’s subjective and objective assessment.	0	0.0	3	6.3	4	8.3	25	52.1	13	27.1	3	6.3
17. Perceived readiness for SCI clinical block when exposed to practical/real-life situations to prepare.	0	0.0	0	0.0	1	2.1	8	16.7	36	75.0	3	6.3
18. Perceived competence to treat PWSCI independently.	2	4.2	3	6.3	15	31.3	17	35.4	8	16.7	3	6.3

SCI, spinal cord injury; PWSCI, people with SCI.

### Differences between socio-demographic information and perceived readiness and competence to treat patients with spinal cord injury

The independent *t*-test was used to determine whether there is a significant difference in self-perceived competence and readiness between students who underwent an SCI clinical block and those who did not. There was a significant difference in self-perceived competence in treating SCI patients between students who had undergone an SCI clinical block and those who had not (*t* [46] = 2.80, *p* = 0.007). There was no significant difference in perceived readiness to treat SCI patients (*t* [46] = 1.82, *p* = 0.075). Similarly, there was a significant difference in self-perceived competence to treat SCI patients when the students had prior experience of treating an SCI patient at the time of data collection (*t* [46] = 2.33, *p* = 0.024), but no significant difference in their readiness (*t* [46] = 1.3, *p* = 0.207).

### Associations between self-perceived competence and readiness and having completed an spinal cord injury clinical block

Table 3 reflects the significant associations between the self-perceived competence and readiness questions and whether students underwent an SCI clinical block and had previously treated a patient with SCI or not. There is a significant association between students’ perception of themselves as competent and ready to treat patients with SCI when they had undergone the SCI clinical block. Furthermore, there was only a significant association between students who had treated PWSCI by the time data were collected and self-perceived competence to treat patients with SCI.

**TABLE 3:** Summary of significant associations from Fisher’s exact test.

Item	SCI block done		Treated PWSCI	
	Fisher’s exact	*p*-value	Fisher’s exact	*p*-value
1. Perceived theoretical knowledge in SCI to manage/treat PWSCI.†	17.20	0.001	-	-
2. Perceived ability to apply theoretical knowledge in SCI practice.†	8.93	0.041	9.24	0.029
3. Perceived ability to deal with the range of medical conditions that may be regarded/classified as SCIs.†	12.99	0.004	12.90	0.005
4. Perceived ability to safely and appropriately perform treatment techniques on PWSCI.†	12.06	0.010	15.57	0.002
5. Perceived ability to perform objective assessments on PWSCI.	15.66	0.001	-	-
6. Perceived ability to prioritise PWSCI’s problems after conducting an assessment.†	-	-	10.77	0.015
7. Perceived ability to identify and set appropriate short- and long-term goals for a PWSCI.†	-	-	9.68	0.026
8. Perceived readiness for SCI clinical block after using online learning resources to prepare.	13.45	0.004	-	-
9. Perceived readiness for SCI clinical block after being assessed on skills using a theoretical patient.	9.92	0.049	-	-
10. Perceived competence to treat PWSCI independently.†	16.85	0.001	10.60	0.030

Note: Significance tested at the level of α < 0.05.

SCI, spinal cord injury; PWSCI, people with SCI.

† , Questions pertain to self-perceived competence.

## Discussion

This study’s demographic picture is similar to that of studies conducted by Talberg and Scott (2014) and Steyl (2011), where final-year physiotherapy students were the target population. Our findings on gender are consistent with those across the physiotherapy profession, as Physiotherapy is a predominantly female profession, except for some countries in the Middle East (Mohammed et al. 2025). According to World Physiotherapy (2022), 86% of physiotherapists in South Africa are female, compared to the global incidence where 63% of physiotherapists are female.

This study found that research articles were the most used resource by physiotherapy students, with textbooks being used the least. Spinal cord injury is an evolving field with numerous articles published monthly by rehabilitative journals. Students can stay up to date with new information in the SCI field in addition to using lecture notes and e-learning websites and apply evidence-based practice. Spinal cord injury textbooks are not prescribed by all universities but rather recommended (Department of Physiotherapy 2020), and this may explain why they are the least used in this population. Cutler (2018) also explains that students are more likely to use textbooks in an online degree, and none of the physiotherapy courses at the universities in South Africa are conducted through an online platform and therefore can further explain why the textbook rating was low.

Our study found high overall perceived competence and readiness rates by physiotherapy undergraduate students. Our findings suggest that the students generally perceive themselves as competent and ready to treat patients with SCI. This is a welcome positive finding, as Varaei et al. (2012) suggested that increased self-perceptions may positively influence clinical performance. These findings also correlate with those of Talberg and Scott (2014), who found that physiotherapy students had high perceived readiness to enter clinical practice. This study found a significant difference in self-perceived competence to treat SCI patients between students who had undergone an SCI clinical block and those who had not, which is unsurprising. This suggests that although students may undergo extensive case study preparation, they would feel more competent in treating a patient with SCI after experiencing it firsthand in a clinical setting. In the study by Recker-Hughes, Neville and Brooks (2015), opportunities for learning essential skills were provided in watching and participating in the simulated encounters. Our findings further support this, as we found that students with prior experience of treating a patient with SCI had higher self-perceived competence in treating SCI patients than those who did not. Students in our study perceived that they had acquired sufficient theoretical knowledge in SCI necessary to treat patients with SCI, irrespective of not completing an SCI clinical block. This suggests that the theoretical SCI component provided was adequate.

Students who completed an SCI clinical block were associated with higher self-perceived competence in dealing with the range of medical conditions and safely and appropriately performing treatment techniques on patients with SCI, as well as treating SCI patients independently. Our findings suggest that students who did not have the opportunity to apply their theoretical knowledge in a clinical setting ‘lose out’ on learning other conditions not focused on as intently as SCI (such as polyneuropathies that may result in lower limb weakness). Peacock and Grande (2015) encourage exposure to different conditions to equip a student with the ability to perform differential diagnoses. Understandably, students who have not completed an SCI clinical block would feel less competent to treat an SCI patient independently, as they would not have had the experience to even treat such a patient under supervision. The use of research articles and other online resources, as well as engaging with written clinical case scenarios, was found to be positively associated with self-readiness in treating an SCI patient in our study. Evidence-based teaching by nursing educators has become an important ideology, as expanding teaching strategies based on recent advances in technology and student learning styles is encouraged (Lauver et al. 2009). Self-perceived readiness to treat SCI patients was also positively associated with performing objective assessments. However, only students with prior experience of treating SCI patients were found to be able to prioritise patients’ functional and impairment problems, as well as set appropriate short-term and long-term goals. This finding suggests that students who have been assessed on and received feedback on their creation of a patient’s problem list and goal development have better competency perceptions because of the possible feedback they received. Bing-You et al. (2017) state that feedback is a crucial means of enhancing learner performance.

### Limitations

As with many online surveys, our study had a low response rate. Online questionnaires were sent out before the outbreak of the coronavirus disease 2019 (COVID-19) pandemic, during which the response period coincided with the time when students were sent home for lockdown. Some students may not have had smartphone access to the online questionnaire and may have relied on school computers to complete the questionnaire. For students with personal laptops, Internet access and data limitations may have hindered participation in our study, as reminders were sent throughout the data collection period. The anxiety and uncertainty of returning to learning and the introduction to online teaching were also considered possible reasons why the students may not have participated in our study. The researchers also had no control over potential internal discussions between participants about the questions in the survey. This could have influenced the responses provided by participants. The self-developed questionnaire used in this study requires psychometric testing, and further validity assessments to ensure that the questions are free from ambiguity. A larger sample size with representation from each university is necessary to generalise these findings. Despite the small sample size, a breakthrough has been established in South African SCI education research.

## Conclusion

Our findings show that clinical exposure and experience in treating patients with SCI are associated with higher perceptions of being able to safely and appropriately perform treatment techniques on patients with SCI. It is widely suggested that wisdom is gained through different experiences and not just by theoretical learning, and our findings support the notion that experience can be the best teacher (Farlex Dictionary of Idioms 2015).
